# Cohort Profile: The Ecuador Life (ECUAVIDA) study in Esmeraldas Province, Ecuador

**DOI:** 10.1093/ije/dyu128

**Published:** 2014-07-02

**Authors:** Philip J Cooper, Martha E Chico, Thomas AE Platts-Mills, Laura C Rodrigues, David P Strachan, Mauricio L Barreto

**Affiliations:** ^1^Laboratorio de Investigaciones FEPIS, Quinindé, Esmeraldas Province, Ecuador,; ^2^Institute of Infection and Immunity Research,; ^3^Institute of Population Health Research, St George’s University of London, London, UK,; ^4^Centro de Investigación en Enfermedades Infecciosas, Escuela de Biología, Pontificia Universidad Católica del Ecuador, Quito, Ecuador,; ^5^Division of Allergy and Clinical Immunology, University of Virginia Health System, Charlottesville, VA, USA,; ^6^Infectious Diseases Epidemiology, London School of Hygiene and Tropical Medicine, London, UK and; ^7^Instituto de Saúde Coletiva, Universidade Federal da Bahia, Salvador, Brazil

## Abstract

The ECUAVIDA birth cohort is studying the impact of exposures to soil-transmitted helminth (STH) parasites and early-life microbial exposures on the development of atopy, allergic diseases and immune responses in childhood. A total of 2404 newborns were recruited between 2006 and 2009 in a public hospital serving the rural district of Quininde, Esmeraldas Province, in a tropical region of coastal Ecuador. Detailed measurements were done around the time of the birth, at 7 and 13 months and at 2 and 3 years, and data collection is ongoing at 5 and 8 years. Data being collected include questionnaires for: sociodemographic, lifestyle, psychosocial (at 4–6 years only) and dietary (at 6–7 years only) factors; childhood morbidity and clinical outcomes; stool samples for parasites; blood samples for DNA, measurements of vaccine responses and other measures of immune function/inflammation; and anthropometrics. Allergen skin prick test reactivity is done from 2 years and measures of airway function and inflammation at 8 years.

**Key Messages**

- Maternal STH infections, the primary study exposure, were observed in 45.9% of mothers during pregnancy.
- The cohort children had a high risk of infection with STH and protozoal parasites during the first 2 years of life: 28.6% of children had at least one documented STH infection.
- An active surveillance sample within the cohort is being used to estimate the burden of infection with a wide range of enteric and respiratory infections during the first 3 years of life. For example, a peak incidence of norovirus of 75.7 infections / 100 person-years was observed between 6 and11 months of age.
- Any episode of eczema by 3 years of age was observed in 17.7% of 2069 cohort children with complete data; 25.9% had wheeze and 17.1% had skin test reactivity to any aeroallergen.
- Immunological analyses have shown that maternal STH infections sensitize the fetal immune response to STH antigens, and that the innate immune response at 2 years does not differ markedly from that observed in affluent countries.

## Why was the cohort set up?

Chronic infections with helminth parasites are associated with the induction of immune tolerance, an effect that may contribute to the regulation of host inflammation and a reduction in the risk of inflammatory diseases such as those associated with allergy.^1^^,^^2^

The most common helminth infections are those caused by the soil-transmitted helminths (STH) including *Ascaris lumbricoides*, *Trichuris trichiura* and hookworm, that are estimated to infect 2 billions worldwide. These parasites are considered to have important effects on nutrition and growth in childhood^3^ and are particularly common among children living in poverty in the warm moist tropics where conditions are optimal for survival and transmission of these parasites through contact with a faecally-contaminated environment.

The apparent low prevalence of atopy and allergic diseases reported from the rural tropics may be explained by the high prevalence of these parasites in children:^1^^,^^4^ although helminth infections can induce allergic-type responses in the human host,^2^^,^^5^ such responses are strongly regulated during chronic infections^2^^,^^6^ and may contribute to a reduction in inflammatory responses to aeroallergens.^6^

Cross-sectional epidemiological studies of schoolchildren have shown an inverse association between the presence of STH infections and the prevalence of skin prick test (SPT) reactivity to aeroallergens.^2^^,^^7^ The effects of STH infections on allergic symptoms are less clear. A meta-analysis of observational studies suggested that hookworm infection was associated with a reduced, but *Ascaris* with an increased, prevalence of asthma symptoms.^8^ Several cross-sectional studies have measured the effects of STH infections on the prevalence of eczema and rhinitis, showing variable effects,^7^ but recent studies have provided little evidence for an association.^9^^,^^10^

Reductions in morbidity caused by STH infections generally require periodic mass treatments with anthelmintic drugs because re-infection rates are high in endemic regions.^11^ Randomized intervention studies of the effects of periodic anthelmintic treatments on allergic outcomes in schoolchildren have provided conflicting evidence, with some showing a post-treatment increase in SPT incidence^12^^,^ or prevalence^13^ whereas others have shown no effect.^14–16^ An analysis of data from communities in Ecuador that had received community-wide anthelmintic treatments for a period of 15 years showed that such treatments were associated with an increase in the prevalence of SPT and eczema in schoolchildren.^17^ Because the intervention was implemented before the children were born, and would have reduced STH exposures during early life (i.e. through a reduction in maternal and early childhood infections), these data could be interpreted to suggest that early exposures are critical for the protective effect.^18^ A randomized controlled study in Uganda showed an increased incidence of eczema to 5 years of age among offspring of mothers treated with anthelmintics during pregnancy.^19^^,^^20^ The protective effects of environmental exposures against allergy appear to be stronger when present in early childhood^21^^,^^22^ or even *in utero*,^23^ such as those associated with farming.

We, therefore, started a birth cohort in a rural district of Ecuador, endemic for STH parasites, to observe the effects of early exposures to STH infections on the development of atopy and allergic disease. The cohort also provides the opportunity to investigate the possible effects of other microbial and environmental exposures in early childhood on the development of atopy and allergic diseases.^24^

## What does the study cover?

The ECUAVIDA cohort is a prospective cohort study from birth, designed to investigate the effects of pre- and postnatal exposures to STH parasites on the development of atopy and allergic diseases to 8 years of age. The study is one of few rural birth cohorts from a developing country—to our knowledge it is the only such cohort being conducted in a rural region of Latin America currently in progress—and provides the opportunity to investigate the role of the hygiene hypothesis more generally through an examination of the effects of a variety of infectious, microbial and environmental exposures on the development of study outcomes. The study population is undergoing the rapid transition from a more traditional rural to an urban way of living and provides the opportunity also to examine the processes associated with this transition on study outcomes. Because diabetes mellitus and cardiovascular and hypertensive disease have emerged as three major causes of death in Ecuadorian adults,^25^ and these outcomes have been associated with chronic low-grade inflammation^26^^,^^27^ and are considered to start in early life,^26–28^ we have included the measurement of clinical indices and biomarkers for these diseases in the cohort to allow us to evaluate the effects on the development of infection and inflammation in early childhood.

The general aims of the project are:

- to measure the impact of prenatal (maternal infections) and postnatal infections with STH parasites on the development of atopy and allergic diseases;
- to explore the effects of other chronic infections and microbial exposures in early childhood on the development of atopy and allergic diseases;
- to explore the effects of chronic infections and microbial exposures on the development of the immune response in childhood, including vaccine immune responses;
- to explore the effects of early-life infections and inflammation on growth and nutritional trajectories during childhood;
- to explore the effects of early-life exposures, both infectious and non-infectious, on the development of allergic diseases and markers for chronic non-communicable diseases (e.g. blood pressure and plasma glucose) during childhood.

## Who is in the cohort?

**Figure 1.**
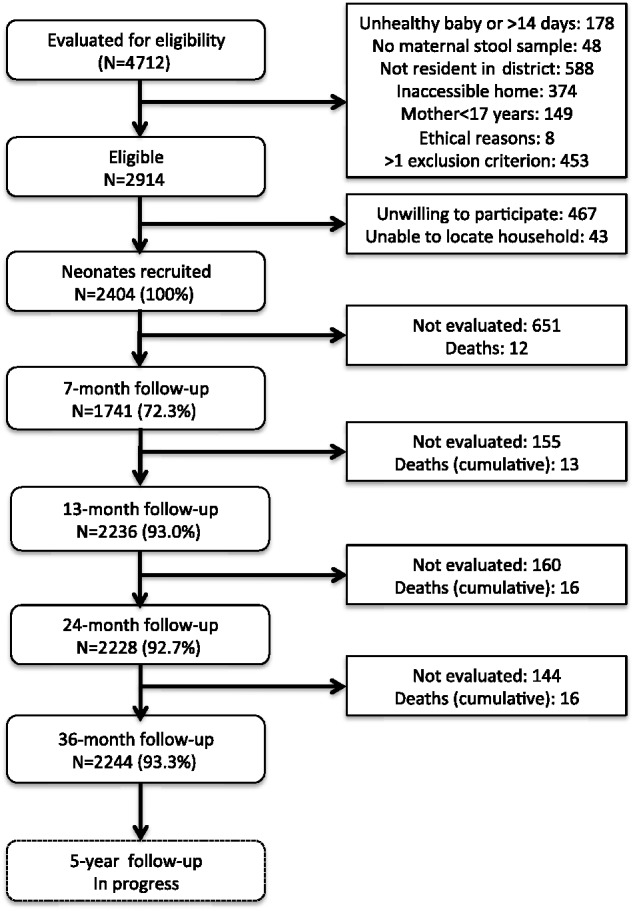
Flowchart illustrating the recruitment and follow-up of the ECUAVIDA cohort to 5 years of age.

**Figure 2.**
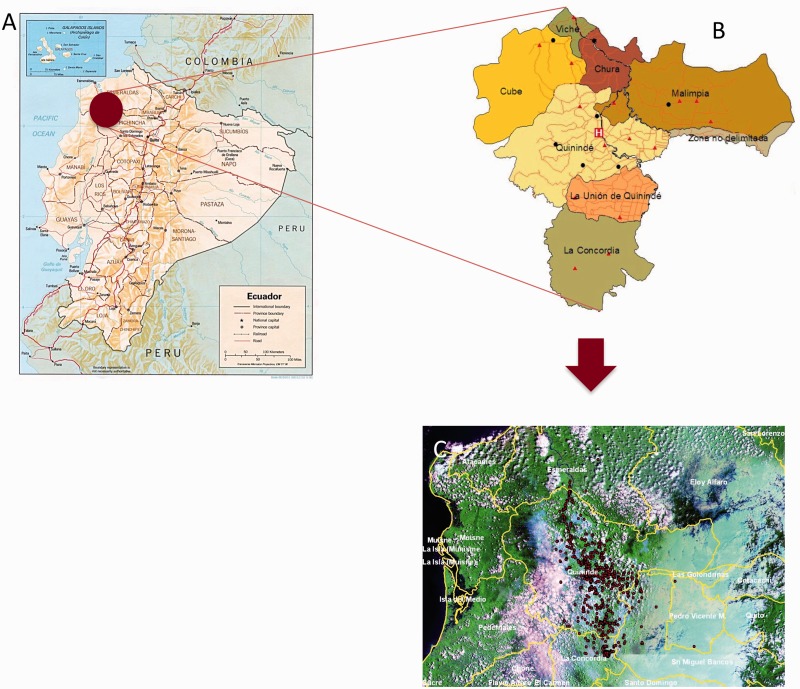
Study site. **(A)** Map of Ecuador showing location of district of Quinindé, Esmeraldas Province (shaded circle) (courtesy of the General Libraries, University of Texas at Austin, TX). The recruitment area for the cohort was defined by the geographical boundaries of this district. **(B)** Map showing parishes the district of Quinindé including La Concordia. H, Hospital Padre Alberto Buffoni. **(C)** geographical location of households of cohort infants.

The study population included eligible newborns delivered at the Hospital Padre Alberto Buffoni (HPAB) between November 2006 and December 2009. HPAB is the only hospital serving the rural district of Quinindé, Esmeraldas Province, Ecuador (Figure 2). During recruitment, members of the study team visited daily the maternity and vaccination departments of HPAB. Inclusion criteria were: (i) healthy baby less than 14 days old; (ii) at least one stool sample collected from the mother in the third trimester of pregnancy or around the time of delivery of the child; (iii) the family has lived in the district of Quinindé for the last 2 years and does not plan to move out of the district over the following 3 years; (iv) the home was accessible; and (v) the family has no ethical or religious principles that might interfere with their participation. Mothers were initially interviewed for eligibility and, if interested and eligible, a home visit was scheduled where informed written consent was obtained and a standardized questionnaire administered. The mothers of 2404 eligible newborns agreed to participate in the study. Reasons for non-participation are shown in Figure 1. The district of Quinindé is primarily dependent economically on agriculture. Within the district are three towns with populations greater than 10 000 inhabitants: Quinindé, La Union and La Concordia. About 70% of the cohort lives in rapidly expanding urban and peri-urban neighbourhoods of these three towns, and the remainder in rural recintos (or settlements). The distribution of study households of cohort children in the district of Quinindé is shown in Figure 2C comparison of available data for Quinindé with data from the cohort, with respect to maternal-child factors and social indicators (Table 3), showed the cohort to be similar with respect to maternal age and ethnicity, and proportion of male births. A comparison of household factors show that the cohort households had greater access to services than the general population in Quinindé, probably a reflection of recruitment at HPAB and differential use of this service between urban and rural populations within the district.

## How are they being followed up?

Data collections are being done at baseline during the initial home visit and at 7, 13, 24, and 36 months and 5 and 8 years of age. Follow-up visits are either scheduled at an HPAB outpatient facility dedicated for the cohort, or through home visits. The study population is highly mobile, largely within the district, and detailed information has been obtained to optimize follow-up—including maps of the location of the household and mobile telephone contacts for the mother and close neighbours.

## What has been measured?

Measurements are done as outlined in Tables 1 and 2. The primary study outcomes are atopy, eczema and asthma. Data on eczema are being collected using standardized instruments based on the UK Working Party (UKWP) criteria/Nottingham protocol^29^ and are measured at 3 years of age. Asthma is measured at 5 and 8 years of age. Atopy is measured as allergen skin test reactivity at 5 years of age. Primary study exposures are the presence of maternal STH infections or presence of STH infections in the child during the first 2 years of life. Standardized questionnaires collect data periodically from the mother on socioeconomic, household and lifestyle factors, demographics, environmental exposures and allergic symptoms in the child—data collected are detailed in Table 1. Physical examinations are done at each observation time. Airways function and reversibility following inhalation of salbutamol, and airways inflammation by FeNO^30^ and cytology in nasal washes will be evaluated at 8 years of age. Routine anthropometric measures include weight (using digital paediatric scales), length and height (using locally made infantometers and stadiometers, respectively) and other measures as listed in Table 2. Blood samples are being collected for routine measurements (haemoglobin and total white cell and differential counts) and plasma (antibodies including IgE) and buffy coats (DNA) are being stored. Peripheral blood leukocytes are also being stimulated with various innate and adaptive immune stimuli to measure cytokine and chemokine responses (Table 2).^31^ Stool samples for detection of enteric parasites are collected from the mother and from the child as shown in Table 2. Samples were also collected from children providing a stool sample at additional sampling times: at 3 (samples from 1294 children), 18 (1020) and 30 (640) months, without active chasing-up of samples. Stool samples for the detection of enteric parasites are examined using a combination of techniques including direct saline mounts, the modified Kato-Katz method, formol-ethyl acetate concentration,^32^ and in samples by carbon coproculture^33^ and real-time polymerase chain reaction (PCR).^34^ An aliquot of stool from each sample is being stored for later analysis of intestinal bacterial microbiota as described.^35^ Nasopharyngeal (NP) swabs have been collected for detection of respiratory viral infections and oropharyngeal swabs for analysis of upper airways bacterial microbiota^36^ at routine observation times and when the child presents to the ECUAVIDA outpatient clinic with respiratory symptoms including wheeze.

**Table 1.** Data collected by questionnaire

Variable	Birth	7 m	13 m	24 m	36 m	5 yrs	8 yrs
Demographics							
Sex of the child	+						
Parental age and ethnicity	+						
Socioeconomic data							
Household income and ownership	+						+
Parental education and occupation	+						+
Household electrical connection/material goods^a^	+						+
Household characteristics							
Household location/urban vs rural	+	+	+	+	+	+	+
Household crowding	+	+	+	+	+	+	+
Sources of drinking water / disposal of faeces	+						+
House construction	+	+					+
Cooking fuels	+						+
Maternal health and disease							
Obstetric history and complications	+						
Chronic diseases including allergic	+						+
Vaccines/antibiotics/medications during pregnancy	+						
Smoking during pregnancy	+						
Child characteristics and exposures							
Mode of birth and related factors	+						
Gestational age^b^	+						
Symptoms of wheezing/eczema/rhinitis		+	+	+	+	+	+
Day care		+	+	+	+	+	
Pet/farming/animal exposures	+	+	+	+	+	+	+
Environmental tobacco smoke	+	+	+	+	+	+	+
Number of siblings	+		+	+	+	+	+
Morbidity including hospitalizations		+	+	+	+	+	+
Use of medications/antibiotics		+	+	+	+	+	+
Vaccination history		+	+	+	+	+	+
Breastfeeding/weaning/diet		+	+	+	+	+	
Allergic symptoms		+	+	+	+	+	+
Psychosocial evaluations^c^						+	
Food frequency questionnaire							+
Father and siblings							
Chronic diseases including allergic	+						+
Urbanization							
Variables to measure urbanization							+

All data are collected from the child’s mother or primary carer.

Mo, months; yrs, years.

^a^Fridge, television, hi-fi, radio.

^b^Estimated from date of last menstrual period.

^c^Data collected with the following instruments: Self Reporting Questionnaire 20 (SRQ-20); Child Behaviour Checklist (CBCL); Perceived Stress Scale (PSS); Medical Outcomes Study Social Support Scale (MOS-SS); Conflict Tactics Scale Parent-child (CTSPC), Community Violence Questionnaire (CVQ).

**Table 2.** Sample collection and clinical and other evaluations

Variable	Birth	3 m	7 m	13 m	18 m	24 m	30 m	36 m	5 yrs	8 yrs
Environmental measures										
*Mattress dust samples*	+^a^			+						
GPS	+^a^									
Clinical measurements										
Child										
Apgar	+^b^									
Clinical examination	+		+	+		+		+	+	+
Weight/height (length)	+		+	+		+		+	+	+
Head and abdominal circumference	+		+	+		+		+	+	+
MUAC/bioimpedance									+	+
Physical activity										+^c^
Evaluation of child allergy										
Clinical examination for flexural dermatitis			+	+		+		+	+	+
Allergen skin prick test reactivity^d^						+		+	+	+
Pulmonary function + reversibility with β_2_ agonist										+
FeNO										+
Nasal wash										+
Sample collection in child										
*Cytokine responses*										
*Innate*	+^e^		+^e^	+^e^		+^e^		+^e^		+^e^
*Adaptive*	+^e^		+	+		+		+	+	+^e^
*Whole blood RNA*	+^e^			+^e^						+^e^
*Cell population studies*	+^e^		+^e^			+^e^				+^e^
*Plasma/Hb/differential/DNA*	+^e^		+	+		+		+	+	+
*Faeces (parasites/DNA)*	+	+^f^	+	+	+^f^	+	+^f^	+	+	+
*Hypo- and nasopharyngeal swabs*	+^e^		+^e^	+^e^		+^e^		+	+	
Mother										
*Plasma/Hb/differential/DNA*	+									
*Cell culture (innate/adaptive)*	+^e^									
*Whole blood RNA*	+^e^									
*Cell populations*	+^e^									
Weight/height/bioimpedance/MUAC									+	+
Allergen skin prick test reactivity^g^		+								
Glucose	+^h^								+	+
Blood pressure									+	+
Faeces (parasites/DNA)	+									
Father										
*Plasma/Hb/differential/DNA*	+									
Allergen skin prick test reactivity^g^		+								
Faeces (parasites)	+									
Weight/height/bioimpedance/MUAC									+	+
Glucose									+	+
Blood pressure									+	+
Household members										
Faeces (parasites)	+									

Italics represent stored samples.

MUAC, mid-upper arm circumference.

^a^Repeated each time there is change of address.

^b^Data available for births in HPAB.

^c^Planned for a sample of the cohort using 7-day triaxial accelerometer.

^d^Saline control, histamine, *Dermatophagoides pteronyssinus/farinae*, American cockroach, fungi mix, mosquito, dog, cat, peanut, milk, egg, and mixed grass pollen.

^e^Sample of 295 children and their mothers for more detailed immunological evaluations.

^f^Passive collections.

^g^Saline control, histamine, *Dermatophagoides pteronyssinus/farinae*, American cockroach, fungi mix, mosquito, dog, cat, *Alternaria, Blomia tropicalis, Chortoglyphus*, and mixed grass pollen.

^h^From antenatal records at HPAB where available.

**Table 3.** Comparison of ECUAVIDA cohort with population in district of Quinindé, Esmeraldas Province, Ecuador, by maternal and child factors and social indicators

Factor	ECUAVIDA Cohort (N = 2404)	District population
Maternal factors		
Maternal age (median)^57^	24 years	26 years
Ethnicity^58^		
Afro-Ecuadorian	25.6%	24.7%
Mestizo	74.0%	72.6%
Indigenous	0.4%	2.7%
Child		
Male births^57^	51.1%	52.6%
Household factors^58^		
House ownership	51.0%	61.1%
Potable water	34.3%	16.0%
Crowding^a^	58.9%	22.1%
Sewage connection	29.8%	10.1%
Electricity	96.0%	83.6%
Cooking materials		
Gas	99.6%	91.6%
Charcoal/wood	2.8%	6.9%

The sample size for district-level information varies by data source.

^a^Crowding defined as >3 people per sleeping room.

Within the cohort, we have done active surveillance with twice-weekly sampling for respiratory tract infections and diarrhoea in a sample of 195 children between birth and 3 years of age. These samples will be analysed for the presence of respiratory viral infections (NP swabs) and enteric viruses and parasites (stools). Cohort children presenting to ECUAVIDA outpatient clinic with fever have been sampled for blood to measure pathogens over 3 calendar years between 2012 and 2014. Dietary consumption of the child is being evaluated using a food-frequency questionnaire when the child is 6–7 years. Questionnaires are being administered to the child’s mother when the child is 4–6 years, to evaluate pyschosocial factors including: (i) Self-Reporting Questionnaire [SRQ-20] developed by WHO^37^ and being used to measure minor psychiatric disorders in the mother;^38^ (ii) Child Behaviour Checklist (CBCL) to assess behavioural problems in the child:^39^^,^^40^ (iii) Perceived Stress Scale (PSS) to measure the perception of stress by the mother;^41^ (iv) Medical Outcomes Study Social Support Scale (MOS-SS) to measure social support of the mother within the community;^42^^,^^43^ (v) Conflict Tactics Scale Parent-Child (CTSPC) to measure psychological and physical maltreatment and neglect of children by parents;^44^ (vi) Community Violence Questionnaire (CVQ) to measure parental exposures to community violence.^45^

## What has it found?

The ECUAVIDA cohort forms part of a Latin American research collaboration called SCAALA (Social Changes, Asthma, and Allergies in Latin America) that includes two prior studies: SCAALA-Salvador^46^ and SCAALA-Esmeraldas.^47^ SCAALA-Salvador is based on a cohort set up in urban neighbourhoods of Salvador, that has been investigating risk factors for allergic diseases within which most analyses have been cross-sectional; whereas SCAALA-Esmeraldas is a cross-sectional study done in Esmeraldas Province, Ecuador, among urban and rural schoolchildren with similar objectives. Many of the methodologies and instruments developed for the SCAALA studies are being used in the ECUAVIDA cohort, and hypotheses generated by these studies are being tested in the ECUAVIDA cohort in a prospective manner using a wider range and/or more refined set of instruments.^45^^,^^48^^,^^49^

Key findings from SCAALA-Esmeraldas to date include the observations that: poor hygiene exposures (e.g. STH infections) were associated with a reduced prevalence of SPT but not of eczema-asthma-rhinitis symptoms; and recent wheeze although reasonably frequent in rural schoolchildren (∼10%), was mild, did not require medication for control and was not strongly associated with atopy (this association was stronger in urban than rural schoolchildren).^10^ STH infections did not protect against allergic symptoms, but allergic sensitization to *Ascaris* was a major risk factor for recent wheeze in rural schoolchildren.^50^ The ECUAVIDA cohort provides the opportunity to understand better the link between STH and other microbial and non-microbial exposures in early childhood, and the development of atopy and allergic diseases.

The ECUAVIDA cohort represents the section of the population of the district that relies on and has access to the public health service and has a relatively low mean household monthly income of US$209, equivalent to the basic monthly wage over the period 2006 to 2009. The majority of mothers had completed primary (58.7%) but not secondary education (26.0%). The population is undergoing a rapid transition from a traditional to a more modern urban lifestyle—approximately half (51.8%) of the mothers had substantial farming exposures during pregnancy, indicating continuing links with a more rural lifestyle, and over half (53.3%) had received at least one course of antibiotics during pregnancy. The primary study exposures were frequent: almost half (45.9%) of the mothers were infected with STH parasites, mainly with *A. lumbricoides* and *T. trichiura*, of which most infections were of light intensity according to WHO criteria.^51^ Infections with hookworm and *S. stercoralis* were of low prevalence among mothers. High rates of infection with STH parasites were observed in cohort children during the first 3 years of life, that is 28.6% of cohort children by 2 years (Table 4) and 42.3% by 3 years.^52^ Maternal STH infections during pregnancy were strongly associated with childhood STH infections, particularly among children of mothers with moderate to high infection intensities with *A. lumbricoides*, and point to a potential and novel intervention strategy for the prevention of STH infections in pre-school children: the targeted treatment of women of childbearing age.^52^

**Table 4.** Prevalence of study exposures and outcomes by 3 years of age in ECUAVIDA cohort children

Exposure	Measure	Outcome
Maternal STH infections (N = 2390)		
Any	% (N)	45.9 (1,098)
*Ascaris lumbricoides*		
Prevalence	% (N)	28.0 (668)
Intensity	GM (range)	683 (35–109, 760)
*Trichuris trichiura*		
Prevalence	% (N)	28.7 (687)
Intensity	GM (range)	22 (35–30, 660)
Hookworm	% (N)	5.8 (139)
*Strongyloides stercoralis*	% (N)	4.0 (96)
Other helminth infections^a^	% (N)	0.5 (13)
STH infections during first 2 years in child (N = 2224)		
Any	% (N)	28.6 (635)
*Ascaris lumbricoides*	% (N)	22.3 (496)
*Trichuris trichiura*	% (N)	12.3 (273)
Hookworm	% (N)	0.4 (9)
*Strongyloides stercoralis*	% (N)	0.8 (18)
Other helminth infections^a^	% (N)	0.9 (20)
Prevalence of study outcomes by 3 years of age		
Eczema (N = 2069)		
Any episode	% (N)	17.7% (367)
Recurrent	% (N)	2.5% (52)
Wheeze (N = 2069)		
Any episode	% (N)	25.9% (536)
Recurrent	% (N)	7.1% (146)
Skin prick test reactivity^b^ (N = 2212)		
Any allergen	% (N)	17.1% (378)
Any aeroallergen	% (N)	15.2% (337)
*D. pteronyssinus/farinae*	% (N)	8.4% (186)
American cockroach	% (N)	3.0% (67)
Fungi	% (N)	2.5% (55)
Dog	% (N)	2.4% (53)
Cat	% (N)	1.1% (25)
Grass	% (N)	2.4% (53)
Any food allergen	% (N)	3.5% (78)
Peanut	% (N)	1.2% (27)
Milk	% (N)	1.2% (27)
Egg	% (N)	1.8% (39)

STH, soil-transmitted helminth; recurrent, 2 or more episodes.

^a^*Hymenolepis* sp.

^b^2-mm cutoff.

With our collaborators, we developed PCR-based diagnostic stools to increase sensitivity for the detection of enteric faecal parasites, both helminth and protozoal, in the cohort. In a random sample of 400 children at 13 months, we detected *Cryptosporidium* spp. in 5.3% of children, *Giardia lamblia* in 31.5%, and *Entamoeba histolytica* in 1.0%.^34^ The corresponding infection rates for the same children at 36 months were 15.4, 45.6% and 2.2%, respectively, indicating high rates of infection with *Cryptosporidium* spp*.* and *G. lamblia* in our study population. Similarly, within the active surveillance sub-sample, we have estimated incidence rates with norovirus infections (GI and GII genotypes) during the first 3 years of life: 0–5 months, 5.2 infections/100 person-years; 6–11 months, 75.7; 12–23 months, 63.8; and 24–36 months, 49.1. Similar analyses are being done for rotavirus infection and the common respiratory viral infections. Such data, in the wider study, will allow us to evaluate if a higher incidence of early childhood infections, either with individual infections or a combination of infections, affect the study outcomes.

Study outcomes to 3 years of age were reasonably common: any episode of eczema was observed in 17.7% and wheeze in 25.9% of 2069 children with complete follow-up data, whereas 17.1% of 2212 children evaluated at 3 years of age had skin test reactivity to any allergen (Table 4). Immunological analyses done in the cohort have provided evidence for *in utero* sensitization to *Ascaris* infections among newborns of mothers with ascariasis: higher frequencies of CD4+ T cells expressing IFN-γ and IL-4 in cord blood from newborns of infected mothers compared with those of uninfected mothers.^53^ Further, cord blood from newborns of infected mothers had higher levels of the immune modulatory cytokine, IL-10, compared with those of uninfected mothers.^54^

Together these data suggest that maternal STH infections can sensitize the foetus *in utero*, presumably through trans-placental transfer of parasite antigens from the maternal circulation, and that such infections are associated with immune regulation, supporting the hypothesis that early STH exposures promote immune regulation in the child. Our observation that maternal STH infections were associated with an increased susceptibility to STH infections in the offspring^52^^,^^54^ implies that such toleration could increase the risk of infection in the child. Regional differences in the risk of inflammatory diseases and immune responses to infections and vaccines could be explained by differences in innate immunity. To determine if innate immune responses in 2-year-old children differ geographically, we have compared innate immune responses to pattern recognition receptor stimuli in a sample of 42 children from the cohort at 2 years of age, with the same responses in 2-year-olds from Belgium, Canada and South Africa.^55^ Innate responses in Ecuadorian children were surprisingly similar to those in Belgian and Canadian children, with only South African children having markedly lower responses overall.^55^ Pyrosequencing comparing the upper airways bacterial microbiota of 24 infants from the cohort with early-onset wheezing, with 24 non-wheezing controls showed a microbiota among wheezers with increased presence of pathogens such as *Haemophilus* and *Staphylococcus* spp.^36^

## What are the main strengths and weaknesses of the study?

A major problem with prospective studies is losses to follow-up. Losses have been complicated by high rates of internal migration within the study area, among a highly mobile population. To date, 63% (1504/2404) children have changed address at least once and some children have changed address up to seven times. However, despite such logistic challenges, we have been able to maintain a high rate of follow-up after 7 months. We were only able to evaluate 72.3% of children at 7 months because of logistic restraints of doing multiple follow-ups simultaneously in a cohort that took 4 years to recruit and where the number of contacts made during the 1st year was probably too frequent for the resources at our disposal. New procedures were put in place to ensure the higher follow-up of 93.0% achieved at 13 months. Such procedures included the setting up a dedicated team for registering changes of address and locating mothers prior to each scheduled follow-up visit, and the collection of more extensive data on family and neighbourhood contacts with mobile telephone numbers. All cohort children had reached 3 years of age by December 2012. Follow-up is shown in Figure 1 and was 93.3% at 3 years of age. Follow-up at 5 years of age is ongoing and the 8-year evaluation will start in 2014. We anticipate a high rate of follow-up at 5 and 8 years of age.

A second limitation was the collection of a maternal stool sample around the time of birth up to 14 days after delivery. At the time of the study, a minority of pregnant mothers attended antenatal clinics at HPAB. We believe that stools collected at this time do represent infections during the third trimester because STH infections are chronic, with adult parasites capable of surviving for several years in the human intestinal tract. Anthelmintic treatment was offered to infected mothers after the birth of the child. We have only limited data on the prenatal period and no measurements of lung function during early childhood.

Another weakness is the reliance on maternal recall for collection of data by questionnaire, which could lead to recall bias because of differential recall depending on maternal age and educational level.

Important strengths are the high rate of follow-up, the large number of repeated measurements and the wide range of infectious and other environmental exposures being measured. The primary exposure of STH infections is being measured using objective, highly specific and reasonably sensitive methods that we have expanded now to more sensitive and specific PCR-based methods in nested samples. The post-neonatal mortality rate observed in the cohort (5.4 deaths/1000 live births) was lower than expected for a low-income setting—the national rate in Ecuador was 10 per 1000 live births in 2011^56^—and probably reflects a combination of recruitment of healthy infants and improved survival by participation in an intensely studied cohort with continuous access to health practitioners.

The study area is a mixture of urban, peri-urban and rural populations, all of which, to a greater or lesser extent, are undergoing urbanization. This should provide valuable insights into the effects of processes associated with urbanization on the development of allergic disease.

## Can I get hold of the data? Where can I find out more?

The data set is not presently freely available but will be made so in the future through an interactive website for the cohort, which will allow interested researchers to make requests for data access. We also welcome specific queries and proposals for collaboration, which should be directed to the corresponding author (pcooper@sgul.ac.uk). Data access is provided on request (pcooper@sgul.ac.uk).

## Funding

The ECUAVIDA cohort is currently funded by the Wellcome Trust [grant 088862/Z/09/Z]. TAEPM is supported by NIH grant AI-20565.
